# Effect of Esophageal Endoscopic Submucosal Dissection on Motility and Symptoms: A Prospective Study

**DOI:** 10.1155/2018/3735473

**Published:** 2018-06-03

**Authors:** Tsutomu Takeda, Kenshi Matsumoto, Akihito Nagahara, Hiroyuki Komori, Yoichi Akazawa, Yuta Nakagawa, Kentaro Izumi, Kohei Matsumoto, Hiroya Ueyama, Yuji Shimada, Daisuke Asaoka, Mariko Hojo, Takashi Yao, Sumio Watanabe

**Affiliations:** ^1^Department of Gastroenterology, Juntendo University School of Medicine, Tokyo, Japan; ^2^Department of Gastroenterology, Juntendo Shizuoka Hospital, Shizuoka, Japan; ^3^Department of Human Pathology, Juntendo University School of Medicine, Tokyo, Japan

## Abstract

**Background:**

Endoscopic submucosal dissection (ESD) of esophageal tumors can cause stenosis, yet the effect of esophageal ESD on motility remains unclarified. This study aimed to compare esophageal motility and symptoms, before and after ESD, using high-resolution manometry (HRM) and symptom scoring.

**Methods:**

Twenty-eight patients with 35 cT1a cancers were prospectively enrolled between December 2014 and February 2016. Pre- and post-ESD symptom score and HRM were recorded. Based on circumferential resection (CR), patients were divided into group A (*n* = 17, <2/3 CR) or B (*n* = 11, 2/3 CR or greater). HRM parameters evaluated were distal contractile integral (DCI), contractile front velocity (CFV), intrabolus pressure, integrated relaxation pressure, distal latency, and peristaltic breaks.

**Results:**

Symptom scores worsened after ESD in 8/11 patients in group B, and 0/17 patients in group A. There was no significant difference in any HRM parameter after ESD in the whole study group but mean DCI tended to increase (*p* = 0.07). In group B, DCI increased significantly after ESD (*p* = 0.04), and CFV tended to decrease after ESD (*p* = 0.08).

**Conclusions:**

DCI tended to increase after esophageal ESD. ESD affected the symptom score and esophageal motility in cases with 2/3 CR or greater. This trial is registered with UMIN000015829.

## 1. Introduction

Endoscopic mucosal resection (EMR) and endoscopic submucosal dissection (ESD) of early esophageal cancers have been performed widely [1–3]. Endoscopic resection of more than approximately 70% of the mucosal defect causes stenosis symptoms [4, 5].

High-resolution manometry (HRM) is a recent development that allows detailed evaluation of esophageal motility disorders. HRM involves esophageal manometry with 36 pressure sensors positioned at 1 cm intervals between the pharynx and the proximal stomach. The pressure data is displayed in color by pressure topography, allowing easy analysis of the internal pressure and peristalsis of the entire esophagus, including the upper esophageal sphincter (UES) and lower esophageal sphincter (LES) [6, 7].

HRM has revealed the physiological function of esophageal motility, and classification of esophageal motility has been renewed accordingly. Pandolfino et al. used HRM to propose a detailed classification system, the Chicago classification, by which esophageal motility disorders are now evaluated [8, 9]. HRM has gained popularity mainly in Europe and the United States; it has been used to evaluate the presence or absence of esophageal motility disorders in many patients, and the Chicago classification was updated in 2012 and 2015 [10, 11]. In Japan, a new HRM system is available that uses a catheter produced by Unisensor AG (Starlet®; Star Medical Inc., Tokyo, Japan), and comparative research with ManoScan® (Sierra Scientific Instruments Inc., Los Angeles, USA) was reported in 2015 [12].

Two previous reports have used HRM to investigate peristaltic movements after esophageal ESD [13, 14], but a pre- and posttreatment comparison was not possible as the study in which the post-ESD peristalsis was evaluated was retrospective. Although HRM was done, there was no review of parameters that could be analyzed by HRM; furthermore, only the dysphagia score was reviewed, while the Eckardt score was not reviewed.

In this study, we evaluated the effect of ESD on esophageal motility and stenosis symptoms by quantitatively evaluating these factors using HRM and symptom scoring before and after ESD.

## 2. Materials and Methods

This was a prospective pilot study performed in a single center in a pre- versus posttreatment design. Written informed consent based on the Helsinki declaration was obtained from all patients. This study protocol was approved by the ethics committee of Juntendo University Hospital (UMIN000015829).

### 2.1. Study Protocol

Patients scheduled to undergo ESD of esophageal cancers in Juntendo University Hospital were prospectively enrolled from December 2014 to February 2016. We included cases determined endoscopically to be cT1a and to be curative with R0 resection pathologically after ESD [15]; cases involving multiple lesions were included when the lesions were treatable on the same day. Intralesional steroid injections (triamcinolone acetonide) [16] were performed to prevent stricture immediately after ESD in accordance with the judgement of endoscopists. Exclusion criteria were (1) advanced cancer and/or lesions ≥ cT1b (lesions thought to involve deep invasion), (2) cases that refused informed consent, (3) patients for whom endoscopic treatment was considered difficult because of serious hepatic, heart, or respiratory diseases, (4) patients in whom a catheter could not be inserted, (5) patients with severe allergy or allergy of unknown cause, (6) patients who needed balloon dilatation due to stenosis after ESD, and (7) patients who needed ESD multiple times on separate days.

### 2.2. Endoscopic Resection and Schedule of Evaluation via Symptom Scoring and High-Resolution Manometry

**Figure 1:**
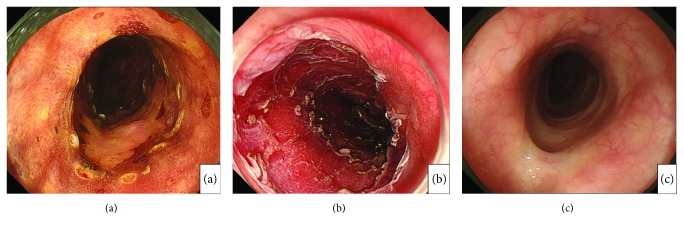
Endoscopic findings in a case of endoscopic submucosal dissection (patient number 20) 0-llb, more than 2/3 circumferential lesion. (a) Chromoendoscopy with iodine staining (with marking around the lesion). (b) Artificial ulcer after 4/5 circumferential resection. (c) Artificial ulcer scar with mild stenosis 8 weeks after endoscopic submucosal dissection.

**Figure 2:**
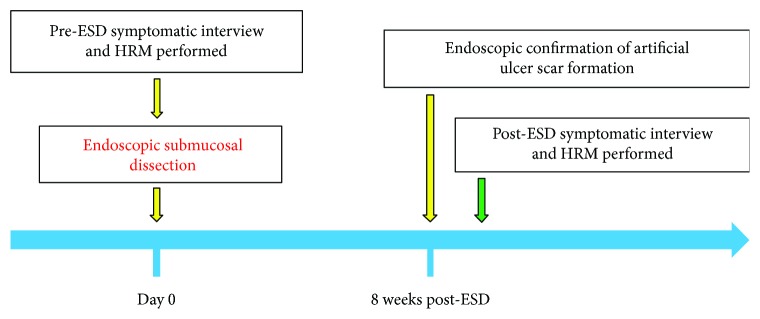
Schedule of symptom scoring, high-resolution manometry (HRM), and endoscopic submucosal dissection (ESD). The patients were interviewed to determine the symptom scores (dysphagia score and Eckardt score) and HRM was performed before ESD. Eight weeks after ESD, the artificial ulcer scar was confirmed endoscopically; the interview to determine the symptom scores and HRM were performed on a separate date to avoid any influence from air insufflation and insertion of the endoscope.

Patients were fasted for at least 12 h before ESD. On the day of treatment, patients were interviewed for symptom score evaluation and HRM was performed before ESD. Eight weeks after ESD, we confirmed endoscopically that the artificial ulcer had changed into scar tissue (Figure 1) [17]; the second HRM test and symptom score interview were then performed on a separate date to the endoscopy in order to minimize the influence of air insufflation and endoscopic insertion on evaluations (Figure 2).

The symptom scores and HRM data were analyzed, the motility function before and after ESD was quantified and combined with the symptom scoring results, and these parameters before and after treatment were compared. As strictures are more likely to occur in cases with more than 2/3 circumferential resection (CR) [4, 5], we divided patients into two groups for analyses: patients who underwent less than 2/3 CR (group A; *n* = 17) and those who underwent 2/3 CR or greater (group B; *n* = 11).

### 2.3. Grading of Symptom Scores

Symptom score was graded according to the Eckardt score [18] for weight loss (0 = none, 1 = <5 kg, 2 = 5–10 kg, and 3= > 10 kg), dysphagia (0 = none, 1 = occasional, 2 = daily, and 3 = each meal), retrosternal pain (0 = none, 1 = occasional, 2 = daily, and 3 = each meal), and regurgitation (0 = none, 1 = occasional, 2 = daily, and 3 = each meal), and the dysphagia score [19] (0 = no dysphagia, 1 = able to eat some solid foods, 2 = able to eat semisolid foods, 3 = able to swallow liquids only, and 4 = unable to swallow anything). The cases were defined as positive if there was a post-ESD increase in even one score compared with the pre-ESD value.

### 2.4. High-Resolution Manometry

**Figure 3:**
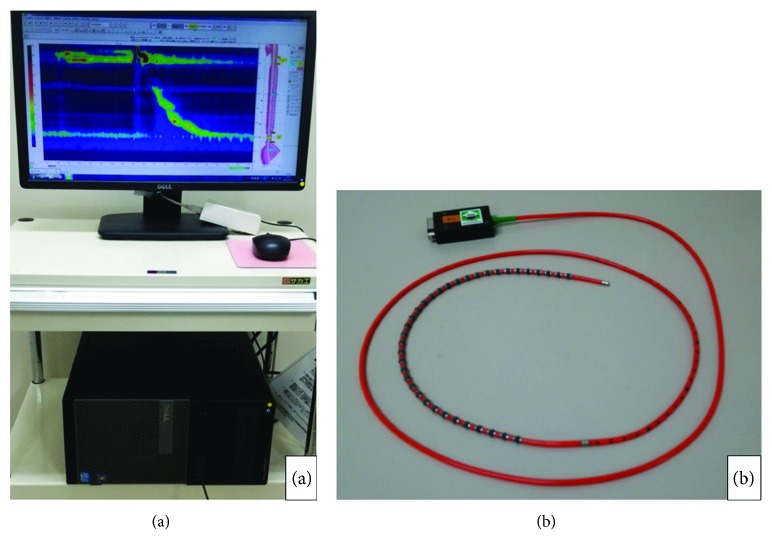
High-resolution manometry (HRM) system. HRM was performed using (a) a Starlet system (Star Medical Inc., Tokyo, Japan) with (b) a catheter (Unisensor AG, Attikon, Switzerland). There were sensor monitors every 1 cm.

Patients were fasted for at least 12 h before HRM. HRM was performed using a Starlet system (Star Medical Inc., Tokyo, Japan) with a catheter (Unisensor AG, Attikon, Switzerland) with the patient in the sitting position (Figure 3). Topical anesthesia was applied to the surface of the nasal mucous membranes before the sensor catheter was inserted. In order to prevent nasal bleeding, 0.05% naphazoline nitrate was applied intranasally, and 4% lidocaine spray was administered twice with a 3 min interval between sprays. Lidocaine (2%) was applied to the catheter, and the catheter was inserted from the external nostril. The high-pressure zones in the UES and LES were checked by the monitor, and the catheter was taped to the nasal alae at the location where both zones could be measured. The patients rested for 10–15 minutes after catheter insertion until the pharyngeal reflex had attenuated. They were then instructed to swallow 5 ml of water on command 10 times, and their esophageal pressure and motility function were measured.

### 2.5. Evaluation of Esophageal Motility

Based on the Chicago classification categories of esophageal motor dysfunction, HRM results were expressed as follows:

- Distal contractile integral (DCI): amplitude × duration × length (mmHg·cm s) of the distal esophageal contraction exceeding 20 mmHg from the transition zone to the proximal margin of the LES
- Contractile front velocity (CFV): slope (cm/s) of the tangent approximation of the 30 mmHg isobaric contour between the proximal pressure trough and the contractile deceleration point (CDP)
- Intrabolus pressure (IBP): the average compartmentalized pressure (mmHg) below each peristaltic contraction in the 5 cm span above the esophagogastric junction (EGJ)
- Integrated relaxation pressure (IRP): mean pressure (mmHg) of the 4 s of maximal deglutitive relaxation in the 10 s window beginning at UES relaxation
- Distal latency (DL): interval (s) between UES relaxation and the CDP
- Peristaltic breaks (PB): gaps (cm) in the 20 mmHg isobaric contour of the peristaltic contraction between the UES and EGJ (measured in axial length)

### 2.6. Esophageal Resection Procedure

The endoscopes used were mainly GIF Q260J (Olympus Tokyo, Japan). A mixture of normal saline with 1% indigo carmine dye was used as the injection solution. However, in the event of poor uptake, an adequate amount of sodium hyaluronate with high viscosity was used. For basic ESD, we performed a precut in the region of the mucosa using a dual knife (KD-650, Olympus Tokyo, Japan) before making a mucosal circumference incision using the dual knife or insulation-tipped knife nano (KD-612L, Olympus Tokyo, Japan). ESD was performed using the insulation-tipped knife nano (dry cut, effect 2, and 30 W, or swift coagulation, effect 4, and 30 W), and/or a dual knife (endo cut 1, effect 2, duration 2, and interval 2, or swift coagulation, effect 3, and 45 W). If there was active bleeding or prominent thick blood vessels were encountered intraoperatively, hemostasis was achieved using coagrasper forceps (FD-410LR, Olympus Tokyo, Japan). A high-frequency surgical unit was used for cutting and coagulation (Erbotom VIO300D; ERBE, Tubingen, Germany).

### 2.7. Definitions

Curative resection was defined according to the expanded criteria of ESD [15] in the case of an R0 and *en bloc* resection. Tumor morphology was described using the Paris classification [20], and pathological findings were described using the Vienna classification [21].

### 2.8. Statistical Analysis

Continuous variables were expressed as the mean ± standard deviation (SD) or median (interquartile range), as appropriate. We used Fisher's exact tests to compare categorical variables, and Student's *t*-test (unpaired) was used for numerical data. *P* values <0.05 were considered significant, and *P* values <0.1 were considered to be tending toward significance. All statistical analyses were performed using SAS statistical package version 9.4 (SAS Institute, Cary, NC, USA).

## 3. Results

### 3.1. Baseline Clinical Results

**Figure 4:**
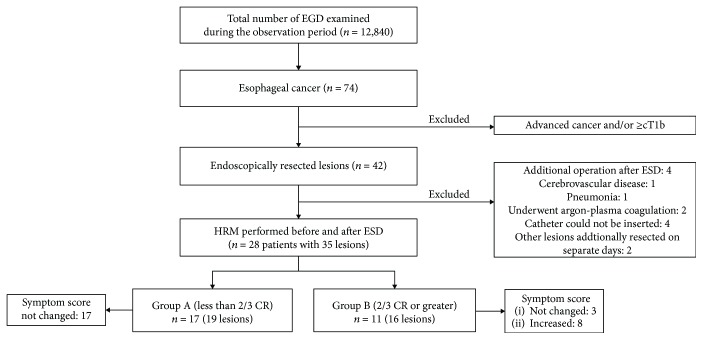
Study outline. Of 42 enrolled patients, 28 patients with 35 lesions were analyzed (group A: *n* = 17, group B: *n* = 11). Eight patients had a post-ESD increase in symptom score; all of these patients were in group B. ESD: esophageal submucosal dissection, cT1b: clinical T1b, HRM: high-resolution manometry, CR: circumferential resection.

The total number of esophagogastroduodenoscopy cases during the study period was 12,840, of which 74 cases were confirmed as esophageal cancer. Advanced esophageal cancer cases in which submucosal deep invasion was suspected endoscopically (≥cT1b) were excluded. Fourteen other patients were excluded for various reasons: four underwent additional surgery after ESD, one had cerebrovascular disease, one had aspiration pneumonia, two underwent Argon-plasma coagulation, four due to the inability to insert the catheter (the nasal catheter could not be inserted in one case, and the catheter could not be inserted in three cases due to strictures after ESD; they then underwent balloon dilatation), and two had other lesions and were additionally resected on separate days (Figure 4). A final total of 28 patients were investigated (male/female 23/5, median age 69.5 years, and range 52–80 years), with 35 lesions acceptable for analysis (Tables 1 and 2). Intralesional steroid injections were performed immediately after ESD on patient numbers 18 and 20 (group B) in accordance with the judgment of endoscopists. We compared patients' backgrounds for these two groups in Table 1: a significant difference in patient backgrounds was not found except for tumor and specimen sizes.

### 3.2. Grading of Symptom Scores

No patients experienced dysphagia after ESD in group A. Patient number 11 in group A occasionally experienced regurgitation but not dysphagia before ESD, but the Eckardt score did not change after ESD. Symptom scores worsened in eight of 11 patients (72.2%) in group B, but in none in group A. In group B, patient number 18 had daily dysphagia; number 21 had 5–10 kg weight loss, daily dysphagia, and daily regurgitation; number 26 had occasional dysphagia and occasional retrosternal pain in the Eckardt score; and numbers 20, 24, 25, and 27 had occasional dysphagia.

### 3.3. High-Resolution Manometry Evaluation

There was no significant difference in any measured HRM parameter after ESD in the entire group (28 cases); however, mean DCI, which indicates the intensity of contraction waves, showed a tendency to increase after ESD (*P* = 0.07; Table 3). There was no significant change after ESD in CFV and DL (which evaluate contraction wave pattern), IBP and IRP (which evaluate EGJ relaxation), or PB (which indicates peristalsis deficit length; Table 3).

**Figure 5:**
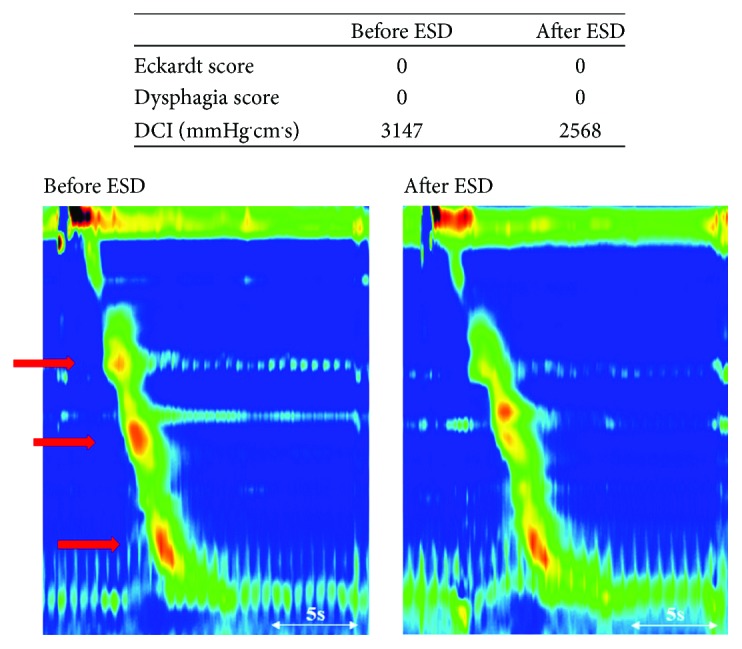
Representative case from group A in which no change was found after endoscopic submucosal dissection (ESD). Patient number 11 is a typical case from group A (less than 2/3 circumferential resection). Symptom score and distal contractile index (DCI) were not increased after ESD. Red arrows indicate physiological contractions: the first, second, and third pressure trough, respectively.

**Figure 6:**
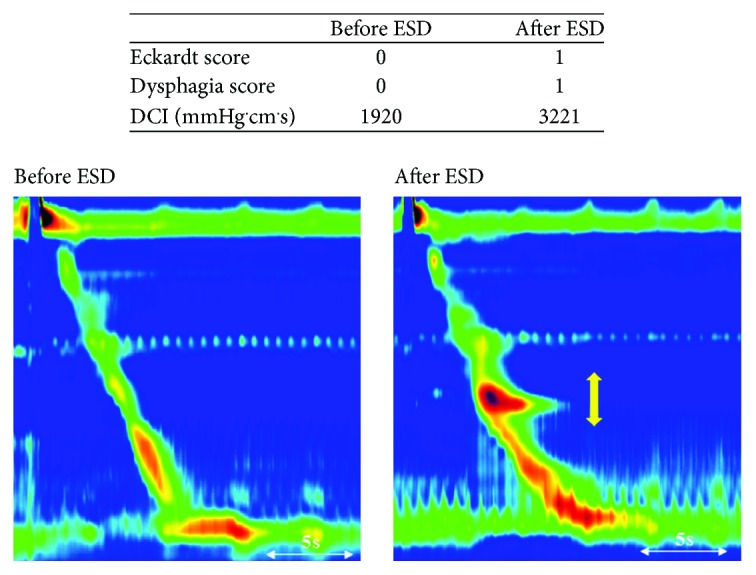
Representative case from group B in which there were changes after endoscopic submucosal dissection (ESD). Patient number 25 is a typical case from group B (more than 2/3 circumferential resection). Symptom score and distal contractile index (DCI) were increased after ESD compared with before ESD. The yellow arrow indicates the resected region (29–32 cm from the incisor column). DCI increased significantly in the resected region.

In group A, there was no significant difference in DCI, CFV, IBP, IRP, DL, and PB before ESD compared with their respective values after ESD (Table 3). In contrast, in group B, DCI increased significantly after ESD (*P* = 0.04), and CFV tended to decrease after ESD (*P* = 0.08); however, IBP, IRP, DL, and PB were not significantly changed after ESD (Table 3). A typical case from group A showed that symptom scores and DCI were not increased after ESD (Figure 5). A typical case from group B showed that symptom scores and DCI increased after ESD (Figure 6).

In group A, only one of 17 cases (5.9%) showed a decrease in CFV after ESD, while 10 of 11 cases (90.9%) in group B showed a post-ESD decrease in CFV. CFV decreased markedly to 5 or more in two patients in group B (numbers 23 and 25). Patient number 23 did not have a post-ESD increase in DCI with a 2/3 CR and there was no post-ESD increase in symptom scores; in contrast, the DCI in patient number 25 with 3/4 CR increased from 1920 to 3221 after ESD and the Eckardt score for dysphagia also increased (Figure 6).

In the group with post-ESD symptom score increases, DCI tended to increase after ESD (*p* < 0.1), but there was no significant change or tendency in other HRM parameters (Table 4). There were also no significant changes in HRM parameters in the group with no post-ESD change in symptom score. All cases in which the symptom score increased after ESD were in group B, and DCI was increased after ESD in six of these eight cases (Table 5).

## 4. Discussion

This is the first prospective study to compare and quantitatively evaluate esophageal motility using HRM before and after esophageal ESD. Bu et al. retrospectively investigated 12 cases using HRM [13]; however, they did not describe the parameters analyzed. In addition, concerning symptoms, only the evaluation of dysphagia was carried out, and a detailed examination using the Eckardt score was not performed. Takahashi et al. investigated cases that had undergone ESD [14]; however, without pretreatment data, the effects of ESD on esophageal motility could not be evaluated, as esophageal peristaltic movement before treatment may not have been normal. Furthermore, this previous report included a case of balloon dilatation, which may potentially have created lacerations in the muscle layer.

In our study, two patients, defined as having weak peristaltic contractions according to the Chicago classification [6], had a pre-ESD DCI value < 450, both of whom were in group B. Patient number 19 had a pre-ESD DCI of 118; post-ESD DCI was similar at 284, and the symptom score also did not increase after ESD. Patient number 28 had a pre-ESD DCI of 449, but this increased to 1583 after ESD and the symptom score also increased. Pre-ESD DCI values in group B were lower compared with group A, but this included the two cases with weak contractions in group B that had DCI < 450. These results indicate that evaluating pre-ESD data is very important.

DCI of all patients tended to increase after ESD (*P* = 0.07) and increased significantly in group B (*P* < 0.05). This may be because esophageal pressure was increased when passing a mildly stenotic site that did not require balloon dilatation. CFV showed no significant change after ESD in all patients, but tended to decrease in group B (*P* = 0.08); it was thought that transit rate slowed down when passing through strictures. This may be because transit velocity decreased due to delayed food passage caused by mild stenosis and reduced esophageal clearance due to post-ESD ulcer scars. IBP and IRP did not significantly change after ESD, and there was no variation in the three cases with lower thoracic lesions. As IBP and IRP are the parameters that evaluate LES sites, they may change post-ESD when junction site lesions like Barret's cancer are resected. Due to the small number of cases of cervical esophagus and upper thoracic esophagus lesions, we cannot draw any conclusions from the present study, but PB may change when these regions are resected. In lesions with less than 2/3 CR, there was no significant post-ESD change in HRM and no post-ESD symptom score increase. Hence, in lesions with less than 2/3 CR, functional impairment does not seem to occur in addition to structural impairment. However, in the present study, lesions in the cervical esophagus and abdominal esophagus were not investigated, which may have altered the outcome. In lesions with 2/3 CR or greater, symptom scores were positive in 72.2% of cases (due to occasional or daily dysphasia, occasional or daily regurgitation, occasional retrosternal pain, or 5–10 kg weight loss). DCI in group B patients increased significantly after ESD. In the eight cases in which the symptom score increased after ESD, DCI also tended to increase (*P* = 0.09), and it is possible that post-ESD change in DCI is involved in the onset of symptoms. In other words, almost all of the cases with 2/3 CR or greater had affected symptom scores and HRM parameters. Further study is required to investigate whether peristalsis regulating drugs are effective in such cases.

Because we evaluated patients before ESD, we were able to clarify the fact that for case number 24, whose dysphagia score greatly increased from 0 to 2, DCI decreased from 1524 pre-ESD to 410 post-ESD; this case was defined as having weak contractions with small breaks and a pre-ESD PB of 4.1. For case number 26, whose Eckardt score increased from 0 to 2, DCI decreased from 837 pre-ESD to 512 post-ESD. Patient number 26 also showed a low DCI before ESD. DCI after ESD for patient numbers 24 and 26 decreased; however, patients experienced dysphagia. The reason for such a discrepancy is thought to be because the extensive artificial ulcer scar may have caused a decrease in esophageal peristalsis in addition to an originally weak esophageal clearance. The Eckardt score for case number 21 increased markedly from 1 to 6. The DCI was relatively low, both before (554) and after ESD (819), with a pre-ESD PB of 3.8. Although the number of cases are limited, these results indicate that when the pre-ESD DCI was low or the pre-ESD PB was high, symptoms may have occurred after ESD because esophageal clearance was originally weak.

Peristalsis of the esophagus has conventionally been evaluated only by an obscure approach, or by symptom score [22–24]. However, the use of HRM has made it possible to quantitatively visualize esophageal motility using a numerical scale, meaning we are now able to objectively evaluate this in greater detail. A few reports have described the evaluation of esophageal motility using HRM after esophageal ESD; however, reports are lacking in which esophageal motility disorders are examined with HRM based on the Chicago classification, before and after esophageal ESD; we are the first to undertake such studies. Our study highlighted a potential for the exacerbation of symptoms after ESD if a peristaltic disorder is observed before esophageal ESD. To date, it has been thought that a stricture and scar in the esophagus after ESD caused an exacerbation of symptoms. In addition to this, patients who originally have a peristalsis abnormality may have a risk of the further exacerbation of symptoms. We thus consider that the factors for symptom exacerbation after ESD can be predicted if peristaltic function can be investigated prior to an ESD procedure.

These results suggest that evaluating pre-ESD HRM data is important. When the pre-ESD DCI is low, a patient may experience dysphagia because of weak esophageal clearance after ESD. Because drug treatment for peristaltic dysfunction can lead to an improvement in symptoms, this gives rise to an expectation of the establishment of a tailor-made drug treatment for patients with dysphagia after ESD. Further study is required to investigate whether modulators of esophageal motility are effective in such cases.

This study had some limitations in that it was carried out in a single center and with a small sample size. Larger studies will enable more detailed analysis, especially in cases where significant differences and tendencies are recognized. We excluded cases of balloon dilatation because of the effect of lacerations in the muscle layer; however, further study is needed in such cases.

In this study, problems were not observed in cases with less than 2/3 CR regarding both symptom score and HRM. Changes were observed after ESD in both symptom score and HRM in cases with 2/3 CR or greater in which balloon dilatation was not performed. In particular, it is thought that worsening symptoms and dysfunction after ESD are likely to appear in cases with weak peristalsis recognized before ESD; further elucidation of disease state and treatment is important.

## 5. Conclusion

Esophageal ESD tended to affect DCI; in particular, it affected both symptom score and esophageal motility in cases with 2/3 CR or greater.

## Figures and Tables

**Table 1:** Baseline characteristics of patients with early esophageal cancers.

Characteristics	All patients (*n*: 28)	Group A (*n*: 17)	Group B (*n*: 11)	*P* value
Sex (males : females)	23 : 5	15 : 2	8 : 3	0.35
Age in years, median (range)	69.5 (52–80)	70.0 (56–80)	67.0 (52–80)	0.71
Morphology				0.92
Protruded (0-I)	0	0	0	
Flat (0-II b, 0-IIb + IIa)	20 (19, 1) (57.1%)	11 (10, 1) (57.9%)	9 (9, 0) (56.3%)	
Depressed (0-IIc)	15 (42.9%)	8 (42.1%)	7 (43.7%)	
Location				0.23
	Ut: 2 (5.7%),	Ut: 2 (10.5%)	Ut: 0	
	Mt: 30 (85.7%)	Mt: 15 (79.0%)	Mt: 15 (93.8%)	
	Lt: 3 (8.6%)	Lt: 2 (10.5%)	Lt: 1 (6.2%)	
Tumor size in mm, median (range)	17 (3–42)	11 (3–40)	25 (12–42)	0.02
Specimen size in mm, median (range)	32 (13–65)	26 (13–50)	39 (30–65)	<0.001
DCI (mmHg·cm·s)	1978 ± 1385	2333 ± 1457	1462 ± 1144	0.11

Ut: upper thoracic, Mt: middle thoracic, Lt: lower thoracic, ESD: endoscopic submucosal dissection, DCI: distal contractile integral. Group A: patients with circumferential resection of less than 2/3. Group B: patients with circumferential resection of 2/3 or greater.

**Table 2:** Overview of patients' clinical information.

No.	Age (years)	Sex	Location	Morphology	Pathology, depth of invasion	Tumor size (mm)	Resected specimen size (mm)	CR	Eckardt score (before/after ESD)	Dysphagia score (before/after ESD)
1	65	M	Ut	0-IIb + IIa	Mod. SCC, EP	7 × 7	23 × 15	1/2	0/0	0/0
2	80	M	Ut	0-IIc	Mod. SCC, LPM	3 × 4	18 × 14	1/5	0/0	0/0
			Mt	0-IIc	Mod. SCC, LPM	5 × 5	22 × 12	1/2		
			Mt	0-IIb	Mod. SCC, LPM	5 × 2	18 × 16	1/5		
3	56	M	Mt	0-IIb	Mod. SCC, LPM	17 × 13	31 × 23	1/2	0/0	0/0
4	68	F	Mt	0-IIb	Mod. SCC, EP	5 × 4	29 × 21	1/2	0/0	0/0
5	77	M	Mt	0-IIb	Mod. SCC, EP	21 × 13	36 × 24	1/2	0/0	0/0
6	59	M	Mt	0-IIb	Mod. SCC, EP	7 × 5	18 × 13	1/4	0/0	0/0
7	60	M	Mt	0-IIb	Mod. SCC, EP	12 × 10	14 × 12	1/5	0/0	0/0
8	56	F	Mt	0-IIb	Mod. SCC, LPM	9 × 10	28 × 25	1/2	0/0	0/0
9	69	M	Mt	0-IIb	Mod. SCC, EP	15 × 13	23 × 19	1/2	0/0	0/0
10	71	M	Lt	0-IIc	Mod. SCC, LPM	20 × 8	39 × 18	1/4	0/0	0/0
11	66	M	Lt	0-IIc	Mod. SCC, LPM	10 × 7	20 × 15	1/5	1/1	0/0
12	79	M	Mt	0-IIc	Mod. SCC, LPM	16 × 10	32 × 25	1/4	0/0	0/0
13	72	M	Mt	0-IIc	Mod. SCC, LPM	10 × 9	22 × 17	1/2	0/0	0/0
14	79	M	Mt	0-IIb	Mod. SCC, LPM	10 × 5	25 × 18	1/5	0/0	0/0
15	70	M	Mt	0-IIc	Mod. SCC, LPM	20 × 15	40 × 25	1/2	0/0	0/0
16	75	M	Mt	0-IIb	Mod. SCC, EP	15× 10	30 × 18	1/2	0/0	0/0
17	80	M	Mt	0-IIc	Mod. SCC, LPM	20 × 15	26 × 15	1/2	0/0	0/0
18	60	M	Mt	0-llc	Mod. SCC, LPM	27 × 35	65 × 39	4/5^∗^	0/2	0/1
19	74	M	Mt	0-llb	Mod. SCC > Por, LPM	42 × 24	58 × 38	3/4	0/0	0/0
20	67	M	Mt	0-llb	Mod. SCC, LPM	35 × 26	47 × 34	4/5^∗^	0/1	0/1
				0-llc	Mod. SCC, LPM	34 × 15	39 × 28	1/2		
21	80	M	Mt	0-llc	Mod. SCC, LPM	25 × 16	43 × 33	2/3	1/6	0/1
22	63	F	Mt	0-llb	Mod. SCC, EP	22 × 16	36 × 31	3/4	0/0	0/0
23	79	M	Mt	0-llb	Mod. SCC, EP	5 × 3	13 × 12	1/5	0/0	0/0
			Mt	0-llb	Mod. SCC, LPM	5 × 17	30 × 13	2/3		
24	72	F	Mt	0-llb	Mod. SCC, EP	25 × 11	39 × 26	3/4	0/1	0/2
			Mt	0-llb	Mod. SCC, LPM	20 × 7	25 × 21	1/2		
25	60	M	Mt	0-llb	Mod. SCC, LPM	40 × 36	46 × 45	3/4	0/1	0/1
26	78	M	Mt	0-llc	Mod. SCC, LPM	33 × 21	47 × 30	2/3	0/2	0/1
27	66	M	Mt	0-llb	Mod. SCC, EP	13 × 20	39 × 26	3/4	0/1	0/1
28	52	F	Mt	0-llc	Mod. SCC, LPM	24 × 15	45 × 30	3/4	0/1	0/1
			Mt	0-llc	Mod. SCC, EP	12 × 9	26 × 15	1/4		
			Lt	0-llc	Mod. SCC, EP	6 × 5	20 × 18	1/4		

Ut: upper thoracic, Mt: middle thoracic, Lt: lower thoracic, Mod. SCC: moderately differentiated squamous cell carcinoma, Por: poorly differentiated squamous cell carcinoma, EP: epithelium, LPM: lamina propria mucosae, CR: circumferential resection, ESD: endoscopic submucosal dissection. ^∗^Intralesional steroid injections (triamcinolone acetonide) were performed immediately after ESD.

**Table 3:** High-resolution manometry data.

	All patients				Group A				Group B		
	Before ESD (mean ± SD)	After ESD (mean ± SD)	*P* value		Before ESD (mean ± SD)	After ESD (mean ± SD)	*P* value		Before ESD (mean ± SD)	After ESD (mean ± SD)	*P* value
DCI (mmHg·cm·s)	1978 ± 1385	2318 ± 1430	0.07		2333 ± 1457	2465 ± 1323	0.57		1462 ± 1144	2104 ± 1615	0.04
CFV (cm/s)	4.8 ± 2.1	4.1 ± 1.6	0.26		4.6 ± 1.9	4.7 ± 2.1	0.92		5.3 ± 2.5	3.6 ± 0.5	0.08
IBP (mmHg)	6.0 ± 6.5	6.3 ± 7.5	0.82		7.2 ± 6.5	8.4 ± 7.6	0.48		4.8 ± 6.7	4.0 ± 6.9	0.65
IRP (mmHg)	10.1 ± 6.9	10.6 ± 7.0	0.72		12.4 ± 7.3	10.9 ± 6.6	0.41		7.5 ± 5.6	10.3 ± 7.7	0.22
DL (sec)	6.9 ± 1.4	7.3 ± 0.8	0.16		7.0 ± 1.0	7.1 ± 0.8	0.79		6.7 ± 1.9	7.6 ± 0.8	0.14
PB (cm)	2.0 ± 1.3	2.0 ± 1.1	0.92		1.6 ± 0.8	1.8 ± 0.8	0.12		2.6 ± 1.5	2.3 ± 1.3	0.19

ESD: endoscopic submucosal dissection, DCI: distal contractile integral, CFV: contractile front velocity, IBP: intrabolus pressure, IRP: integrated relaxation pressure, DL: distal latency, PB: peristaltic breaks, group A: patients with circumferential resection of less than 2/3, group B: patients with circumferential resection of 2/3 or greater.

**Table 4:** High-resolution manometry data for the group showing increases in symptom scores after endoscopic submucosal dissection.

	Before ESD (mean ± SD)	After ESD (mean ± SD)	*P* value
DCI (mmHg·cm·s)	1614 ± 1258	2255 ± 1717	0.09
CFV (cm/s)	5.0 ± 2.3	3.7 ± 0.5	0.17
IBP (mmHg)	5.9 ± 7.3	4.8 ± 7.1	0.62
IRP (mmHg)	7.2 ± 6.3	9.3 ± 8.2	0.39
DL (sec)	6.5 ± 1.7	7.4 ± 0.9	0.12
PB (cm)	2.3 ± 1.1	2.1 ± 1.2	0.45

All cases were from group B (with circumferential resection of 2/3 or greater). DCI: distal contractile integral, CFV: contractile front velocity, IBP: intrabolus pressure, IRP: integrated relaxation pressure, DL: distal latency, PB: peristaltic breaks.

**Table 5:** Review of patients with increases in symptom scores after endoscopic submucosal dissection.

No.	Age (years)	Sex	Location	Morphology	Pathology, depth of invasion	Tumor size (mm)	Resected specimen size (mm)	CR	Eckardt score (before ESD/after ESD)	Dysphagia score (before ESD/after ESD)	DCI before ESD	DCI after ESD
18	60	M	Mt	0-llc	Mod. SCC, LPM	27 × 35	65 × 39	4/5	0/2	0/1	2201	3736
20	67	M	Mt	0-llb	Mod. SCC, LPM	35 × 26	47 × 34	4/5	0/1	0/1	4319	5363
				0-llc	Mod. SCC, LPM	34 × 15	39 × 28	1/2				
21	80	M	Mt	0-llc	Mod. SCC, LPM	25 × 16	43 × 33	2/3	1/6	0/1	554	819
24	72	F	Mt	0-llb	Mod. SCC, EP	25 × 11	39 × 26	3/4	0/1	0/2	1520	410
			Mt	0-llb	Mod. SCC, LPM	20 × 7	25 × 21	1/2				
25	60	M	Mt	0-llb	Mod. SCC, LPM	40 × 36	46 × 45	3/4	0/1	0/1	1920	3221
26	78	M	Mt	0-llc	Mod. SCC, LPM	33 × 21	47 × 30	2/3	0/2	0/1	837	512
27	66	M	Mt	0-llb	Mod. SCC, EP	13 × 20	39 × 26	3/4	0/1	0/1	1087	2598
28	52	F	Mt	0-llc	Mod. SCC, LPM	24 × 15	45 × 30	3/4	0/1	0/1	449	1583
			Mt	0-llc	Mod. SCC, EP	12 × 9	26 × 15	1/4				
			Lt	0-llc	Mod. SCC, EP	6 × 5	20 × 18	1/4				

CR: circumferential resection, Mt: middle thoracic, Lt: lower thoracic, Mod. SCC: moderately differentiated squamous cell carcinoma, EP: epithelium, LPM: lamina propria mucosae, DCI: distal contractile integral (>20.0 mmHg·cm·s).
